# A Neonatal Mouse Model for Pressure Overload: Myocardial Response Corresponds to Severity

**DOI:** 10.3389/fcvm.2021.660246

**Published:** 2021-05-21

**Authors:** Jielei Gu, Xuke Chen, Yangshuo Jin, Mingke Liu, Qiong Xu, Xiaolin Liu, Zhenyu Luo, Sisi Ling, Ningning Liu, Shiming Liu

**Affiliations:** Guangdong Key Laboratory of Vascular Diseases, State Key Laboratory of Respiratory Disease, Guangzhou Institute of Cardiovascular Disease, The Second Affiliated Hospital of Guangzhou Medical University, Guangzhou, China

**Keywords:** neonatal mouse, pressure overload, cardiomyocyte proliferation, extracellular matrix, myocardial hypertrophy

## Abstract

The heart regeneration after apical resection and myocardial infarction in neonatal mice has been studied for years. However, the response of neonatal mouse heart under pressure overload is seldom explored. This study aimed to induce pressure overload in neonatal mice through a transverse aortic constriction (TAC) with different-gauge needles so as to investigate the effect of pressure overload on cardiomyocyte proliferation and hypertrophy in these mice. Myocardial hypertrophy was evaluated by echocardiographic, pathological, and molecular analyses. Cardiomyocyte proliferation was detected by immune-staining of phospho-histone H3, Ki67, and 5-bromo-2-deoxyuridine. Mild pressure overload induced with a 30-gauge needle stimulated cardiomyocyte proliferation, adaptive hypertrophy, and angiogenesis. The heart function was not hampered even 21 days after the surgery. Moderate pressure overload induced with a 32-gauge needle led to pathological myocardial hypertrophy, fibrosis, and heart failure 7 days after the surgery. The gene and protein expression levels of markers of hypertrophy and fibrosis increased in 32-gauge TAC group compared with that in sham and 30-gauge TAC groups. The mice barely survived after severe pressure overload induced with a 34-gauge needle. The findings of this study might provide new insights into cardiomyocyte proliferation and hypertrophy in neonatal mice under pressure overload.

## Introduction

Cardiovascular disease is the leading cause of death globally, affecting patient well-being (1, 2). Since 2011, numerous studies have shown that heart regeneration does occur after myocardial infarction (MI), apical resection, and cryoinjury in neonatal mice compared with adults (3–5). Most studies indicate that neonatal heart recovers functional myocardium within 4 weeks without noticeable scarring (6). The proliferation of preexisting cardiomyocytes is an innovative way of heart regeneration within a short time window after birth (7). However, the regenerative capacity is lost after postnatal day 7 (8, 9). Researchers aim to discover relative factors that promote cardiac repair in neonates to guide heart injury treatment in adults (10, 11). Transverse aortic constriction (TAC) is a well-accepted pressure overload model that induces myocardial hypertrophy and fibrosis in adult mice and rats (12–14). It causes evident myocardial remodeling and systolic and diastolic cardiac dysfunction, eventually resulting in heart failure (15, 16). Compared to MI in neonates, congenital diseases with increased cardiac afterload are more common. Coarctation of the aorta (CoA) is one of the congenital disabilities accounting for 4–8% of congenital heart defects (17).

TAC models in adult mice and rats have been studied for decades. However, whether cardiomyocytes can be stimulated to proliferate under pressure overload in neonatal mice was seldom explored. In 2017, researchers found that pulmonary artery banding in neonatal rats caused right ventricular hypertrophy 7 days after the surgery (18). Meanwhile, the other team found that ascending aortic constriction in neonatal rats promoted cardiomyocyte proliferation (19). Further, neonatal mouse transverse aortic constriction in the regenerative phase induced a positive response with normal heart function, while myocardial fibrosis and hypertrophy developed when TAC was performed in the nonregenerative phase (20). However, whether cardiomyocytes of neonates can be stimulated under pressure overload to proliferate or undergo hypertrophy has not reached a consensus. One possible reason is that the degree of pressure overload led to variable outcome.

Hence, the present study was performed to investigate whether these distinct consequences resulted from different pressure overload levels. A TAC model was established with a 30-gauge (30G), 32-gauge (32G), or 34-gauge (34G) needle, which represented mild, moderate, and severe pressure overload, respectively. It helps better understand how the heart respond to different degrees of pressure overload and choose different constriction according to the research purpose in future. The change in cardiac homeostasis also had promising clinical implications for treating patients with CoA.

## Results

### Mice Growth Was Affected by the Degree of Constriction

**Figure 1:**
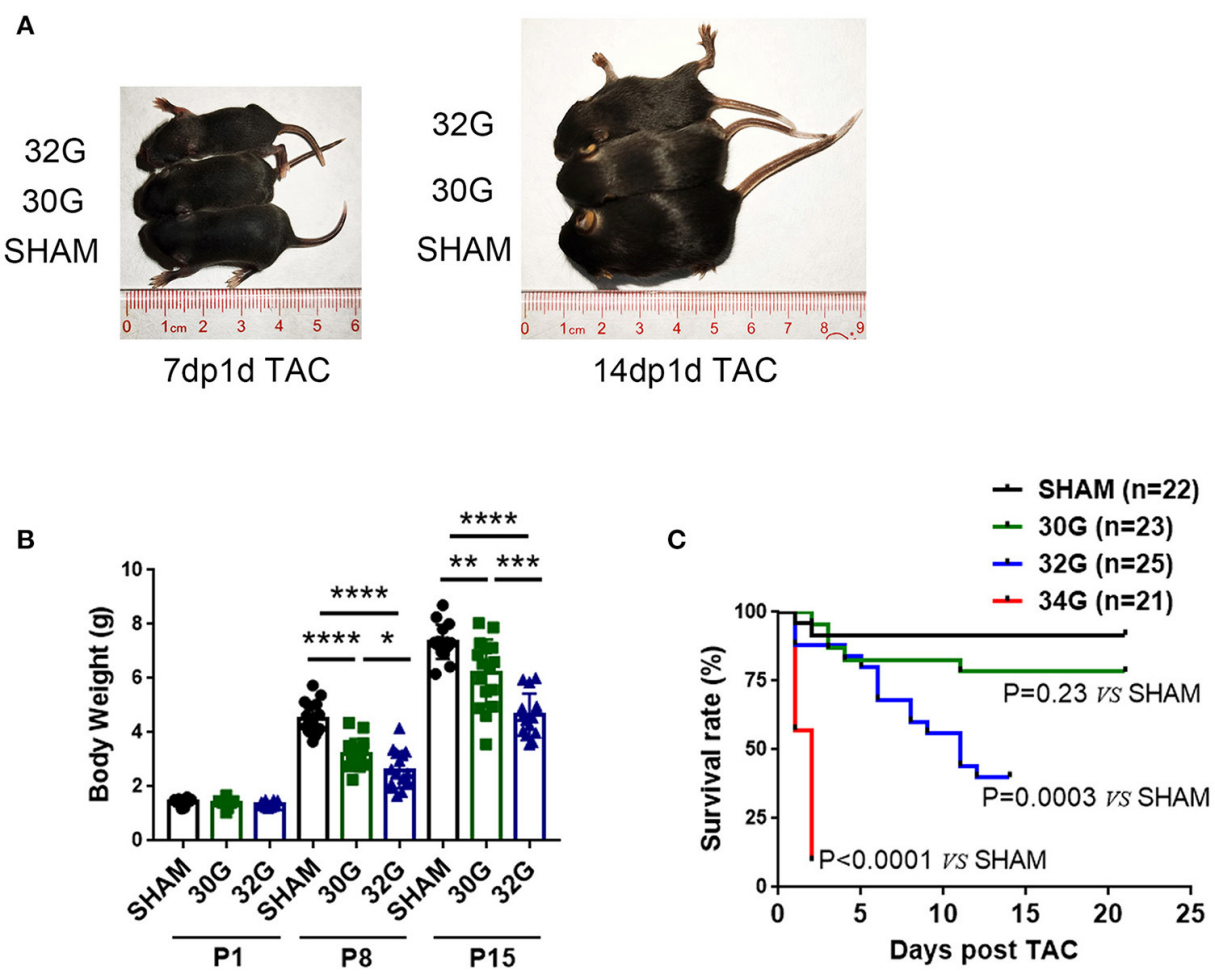
Effect of different degrees of pressure overload on the growth of neonatal mice. **(A,B)** Both 30-gauge (30G) and 32-gauge (32G) TAC affected the growth and development of mice; Representative images of 32G TAC mice (up), 30G TAC mice (middle) and sham mice (down); 7dp1d TAC: 7 days post 1 day TAC; 14dp1d TAC: 14 days post 1 day TAC; P1: postnatal day 1; P8: postnatal day 8; P15: postnatal day 15; *n* (number of mice) = 10, P1 sham; *n* = 10, P1 30G TAC; *n* = 10, P1 32G TAC; *n* = 15, P8 sham; *n* = 15, P8 30G TAC; *n* = 16, P8 32G TAC; *n* = 16, P15 sham; *n* = 16, P15 30G TAC; *n* = 15, P15 32G TAC. **(C)** Kaplan–Meier survival curve of sham, 30G TAC, 32G TAC, and 34-gauge (34G) TAC groups; *n* = 22, sham*; n* = 23, 30G TAC; *n* = 25, 32G TAC; *n* = 21, 34G TAC. The survival rate of the four groups was assessed by Kaplan–Meier analysis and a log-rank test. Data are presented as mean ± SEM. *P < 0.05, **P < 0.01, ***P < 0.001, and ****P < 0.0001. Statistical significance was calculated using the unpaired two-tailed *t*-test.

The 30G, 32G, and 34G TAC surgeries were performed to investigate the myocardial response to different pressure overload levels in neonatal mice. The more severe the constriction, the slower the growth of the mice (Figures 1A,B). Both 30G and 32G TAC reduced the body weight of mice on postnatal day 8 (P8) and postnatal day 15 (P15) compared with that of sham mice. Mice in the 34G TAC group died early after the surgery, while those in the 30G TAC group had a higher survival rate (Figure 1C). The experiment was not continued due to high mortality among pups in the 34G TAC group. More than half of 32G TAC mice died 14 days after the surgery. Therefore, they were sacrificed on P15, and a long-term study was not performed. The surgery was evaluated by subsequent echocardiography.

### 32G TAC Aggravated Cardiac Dysfunction but 30G TAC Did Not

**Figure 2:**
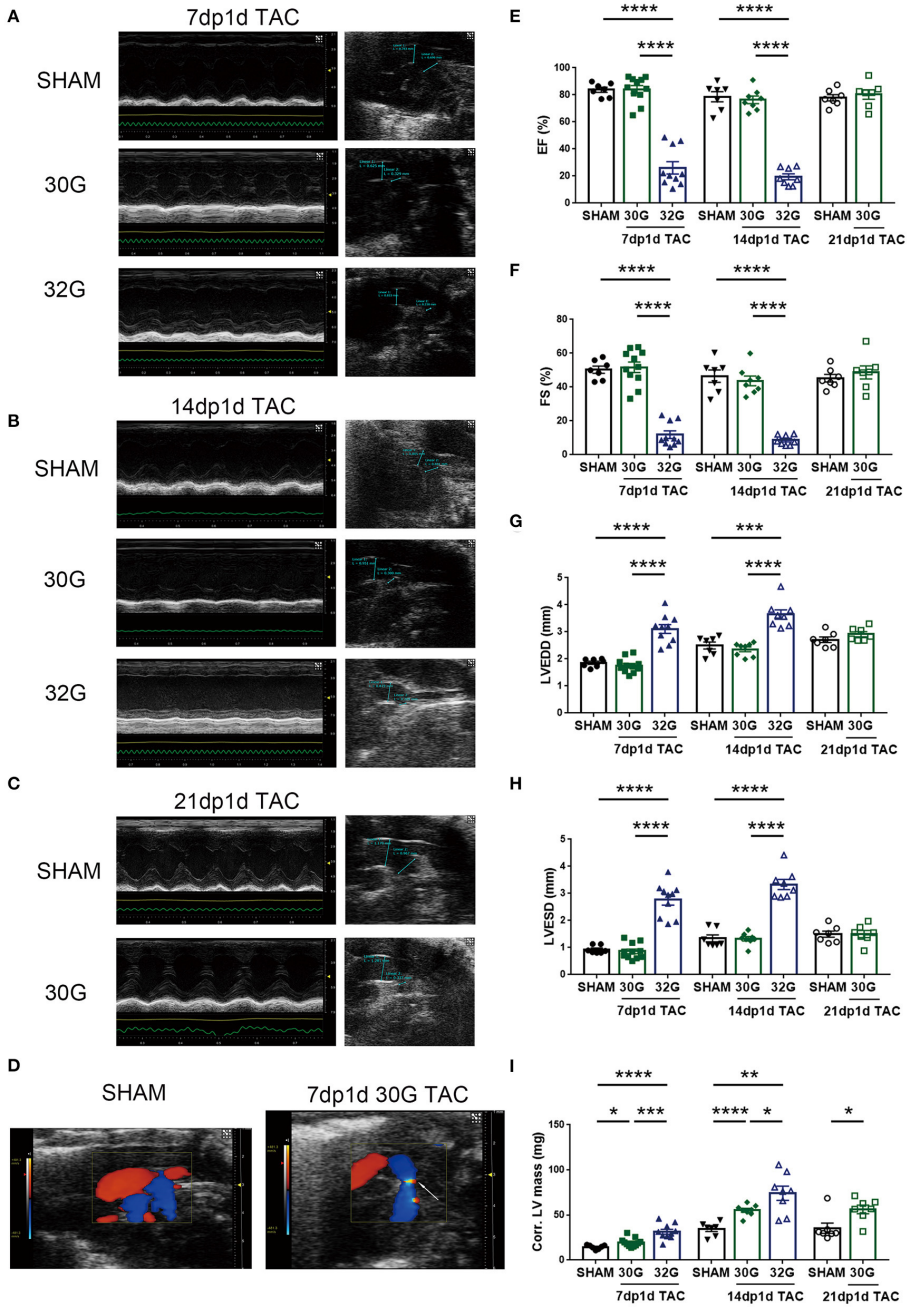
Middle pressure overload significantly impaired heart function, while mild pressure overload did not. **(A,B)** Representative M-mode echocardiographic and aortic arch images of mice in the sham, 7dp1d TAC, and 14dp1d TAC groups with 30G and 32G constriction. **(C)** Representative images of M-mode echocardiograph and aortic arch in sham and 21 days post 1 day TAC (21dp1d TAC) groups with 30G constriction. **(D)** Color Doppler mode images of aortic arch blood flow in sham and TAC groups after 7 days of surgery. The arrow points to the turbulent blood flow area. **(E–I)** Ejection fraction (EF), fractional shortening (FS), left ventricular end-diastolic dimension (LVEDD), left ventricular end-systolic dimension (LVESD), and corrected left ventricular mass (Corr.LV mass) were assessed by echocardiography; *n* = 7, 7dp1d sham; *n* = 11, 7dp1d 30G TAC; *n* = 10, 7dp1d 32G TAC; *n* = 7, 14dp1d sham; *n* = 8, 14dp1d 30G TAC; *n* = 8, 14dp1d 32G TAC; *n* = 7, 21dp1d sham; *n* = 7, 21dp1d 30G TAC. Data are presented as mean ± SEM. *P < 0.05, **P < 0.01, ***P < 0.001, and ****P < 0.0001. Statistical significance was calculated using the unpaired two-tailed *t*-test.

The left ventricular (LV) structure, systolic function, and aortic arch constriction degree were evaluated by echocardiography to explore the effects of different pressure overloads on cardiac function. The aortic arch diameter decreased with the increasing degree of constriction (Figures 2A,B). The pressure load of the left ventricle is positively correlated with the degree of constriction (21). Color Doppler mode images displayed laminar blood flow in the sham group and turbulent blood flow in the TAC group 7 days after the surgery (Figure 2D).

The TAC significantly affected the LV motion 7 days after the surgery. A prominent thickened ventricular wall and narrowed ventricular chambers were found in the M-mode echocardiography of mice subjected to 30G TAC (Figure 2A). The elevation of LV posterior wall thickness during diastole (LVPW diastole) was consistent with concentric hypertrophic features (Supplementary Table 1). Mice in the 32G TAC group had an enlarged ventricular cavity and diastolic LVPW showed no noticeable change, following the characteristics of eccentric hypertrophy (Figure 2A and Supplementary Table 1).

A 30G constriction did not damage the heart function of mice in the 7dp1d TAC subgroup (Figures 2E–H and Supplementary Table 1). The ejection fraction (EF) and fractional shortening (FS) were not affected by mild pressure overload. On the contrary, EF and FS were reduced by an average of 68.1 and 76.3% in the 7dp1d TAC subgroup subjected to 32G constriction, respectively, compared with the sham mice. Additionally, LV end-diastolic volume and LV end-systolic volume increased 3.7 and 17.5 times compared with that in the sham mice, respectively (Supplementary Table 1). The data showed that mice in the 32G TAC group had severe heart failure only 7 days after the surgery, much earlier than the heart failure in adult mice after TAC. Echocardiography detected a 26.5 and 103.4% increase in the corrected LV mass in the sham group compared with the 30G and 32G TAC groups, respectively (Figure 2I).

The EF and FS showed a more deteriorated heart function 14 days after 32G TAC; their averages dropped to 19.49 and 10.11%, respectively (Figures 2E,F). Additional heart function impairments, such as reducing cardiac output and stroke volume, were noted (Supplementary Table 2). Meanwhile, the heart function of mice in the 30G TAC group was not significantly affected. Moreover, a normal cardiac function was maintained in the 30G TAC group 21 days after the surgery (Figures 2C,E–H and Supplementary Table 3). Myocardial hypertrophy was found to be increased 14 days after TAC. The average corrected LV mass increased by 59.6% and 114.2% in the 30G and 32G TAC groups, respectively, compared with the sham group (Figure 2I).

### Requirement of an Appropriate Pressure Overload for Enhancing Cardiomyocyte Proliferation and Angiogenesis

**Figure 3:**
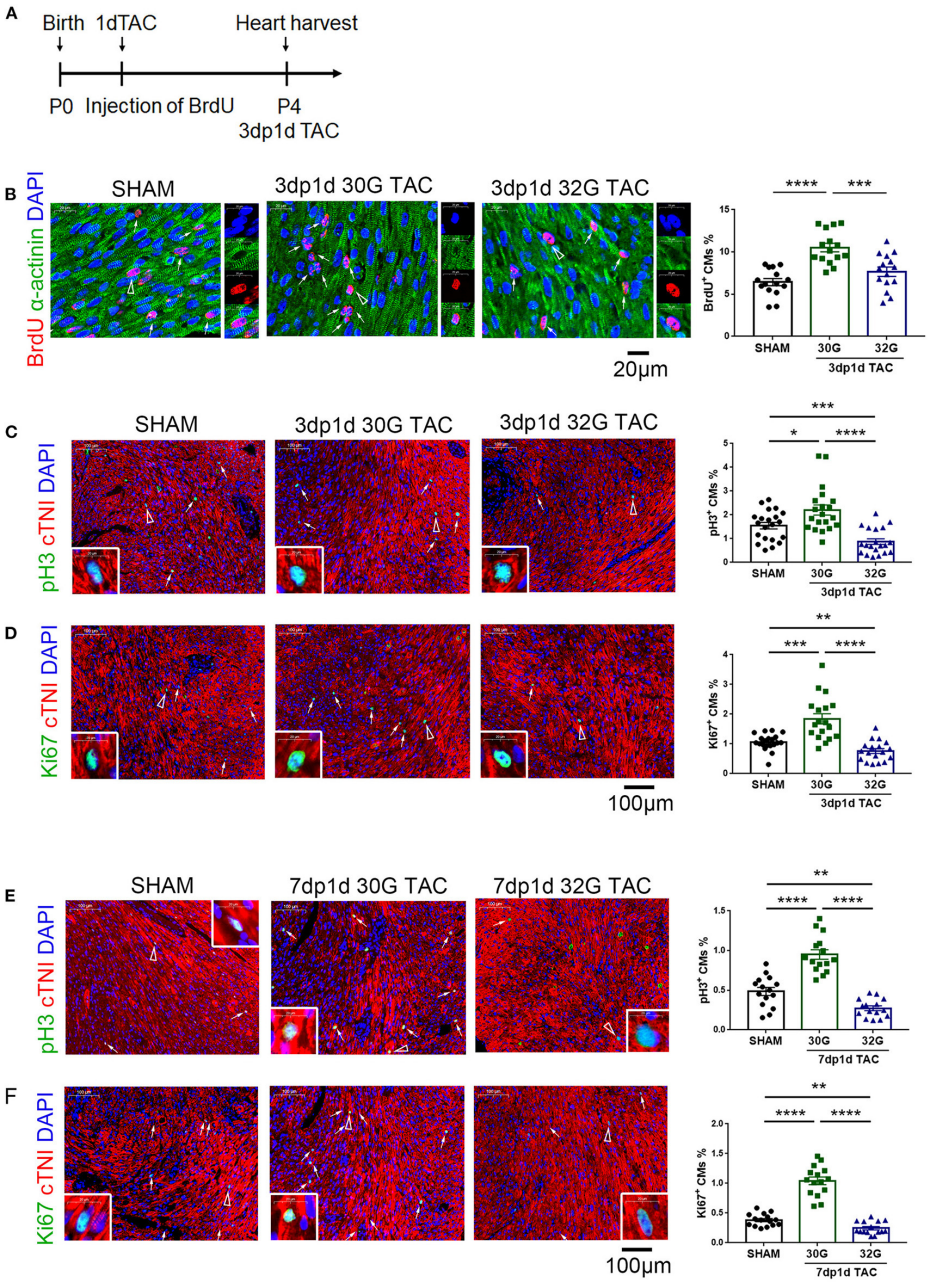
Cardiomyocyte proliferation increased in the hearts of mice in the 30G TAC group but not in the 32G TAC group. **(A)** Timeline of 5-bromo-2-deoxyuridine (BrdU) injection and detection. 1dTAC: TAC performed on postnatal day 1; 3dp1d TAC: 3 days post 1 day TAC. **(B)** The nascent cardiomyocytes were identified by BrdU (red), 4′,6-diamidino-2-phenylindole (DAPI, blue) and α-actinin (green) co-localization in the hearts of mice in the sham, 30G TAC, and 32G TAC groups 3 days after TAC. Scale bar: 20 μm; *n* = 3 hearts per group. Each heart slide was randomly selected with five high-power fields for statistical analysis. **(C,D)** Immunostaining for phospho-histone H3 (pH3, green) and cardiac troponin I (cTNI, red) co-localization and Ki67 (green) and cTNI (red) co-localization to recognize cardiomyocyte proliferation in the heart sections of mice in the sham, 30G TAC, and 32G TAC groups 3 days after TAC. Scale bar: 100 μm; *n* = 3 hearts per group. Each heart slide was randomly selected with five to seven high-power fields for statistical analysis. **(E,F)** Cardiomyocyte proliferation was recognized by immunostaining for pH3, Ki67, and cTnI in the heart sections of mice in the sham, 30G TAC, and 32G TAC groups 7 days after TAC. Scale bar: 100 μm. Each heart slide was randomly selected with four to six high-power fields for statistical analysis. In panel **(A)**, BrdU^+^ cardiomyocytes (triangle arrow) are shown on the right in high-power field images. In panels **(C–F)**, arrows point to pH3^+^ or Ki67^+^ cardiomyocytes. A pH3^+^ or Ki67^+^ cardiomyocyte is indicated by a triangular arrow on the insert image. Data are presented as mean ± SEM. *P < 0.05, **P < 0.01, ***P < 0.001, and ****P < 0.0001. Statistical significance was calculated using the unpaired two-tailed *t*-test.

It was hypothesized that appropriate pressure load might attribute to cardiomyocyte proliferation to investigate why 30G TAC maintained a normal heart function while 32G TAC led to a heart failure. For this, 5-bromo-2-deoxyuridine (BrdU), phospho-histone H3 (pH3), and Ki67 immunostaining was performed together with α-actinin or cardiac troponin I (cTnI) to identify cardiomyocyte proliferation in the hearts of mice in the sham, 30G, and 32G TAC groups. The results showed that the BrdU^+^ cardiomyocytes increased in the hearts of mice in the 30G TAC group 3 days after TAC (Figures 3A,B). The number of pH3^+^ and Ki67^+^ cardiomyocytes also increased in the hearts of mice in the 30G TAC group compared with that in the sham group 3 and 7 days after TAC (Figures 3C–F). On the contrary, the number of BrdU^+^ cardiomyocytes had no evident change in the hearts of mice in the 32G TAC group compared with that in the sham group, but it significantly decreased compared with that in the 30G TAC group (Figure 3B). The positive staining for pH3 and Ki67 in the hearts of mice in the 32G TAC group demonstrated an evident decrease compared with that in the sham and 30G TAC groups 3 and 7 days after TAC (Figures 3C–F).

**Figure 4:**
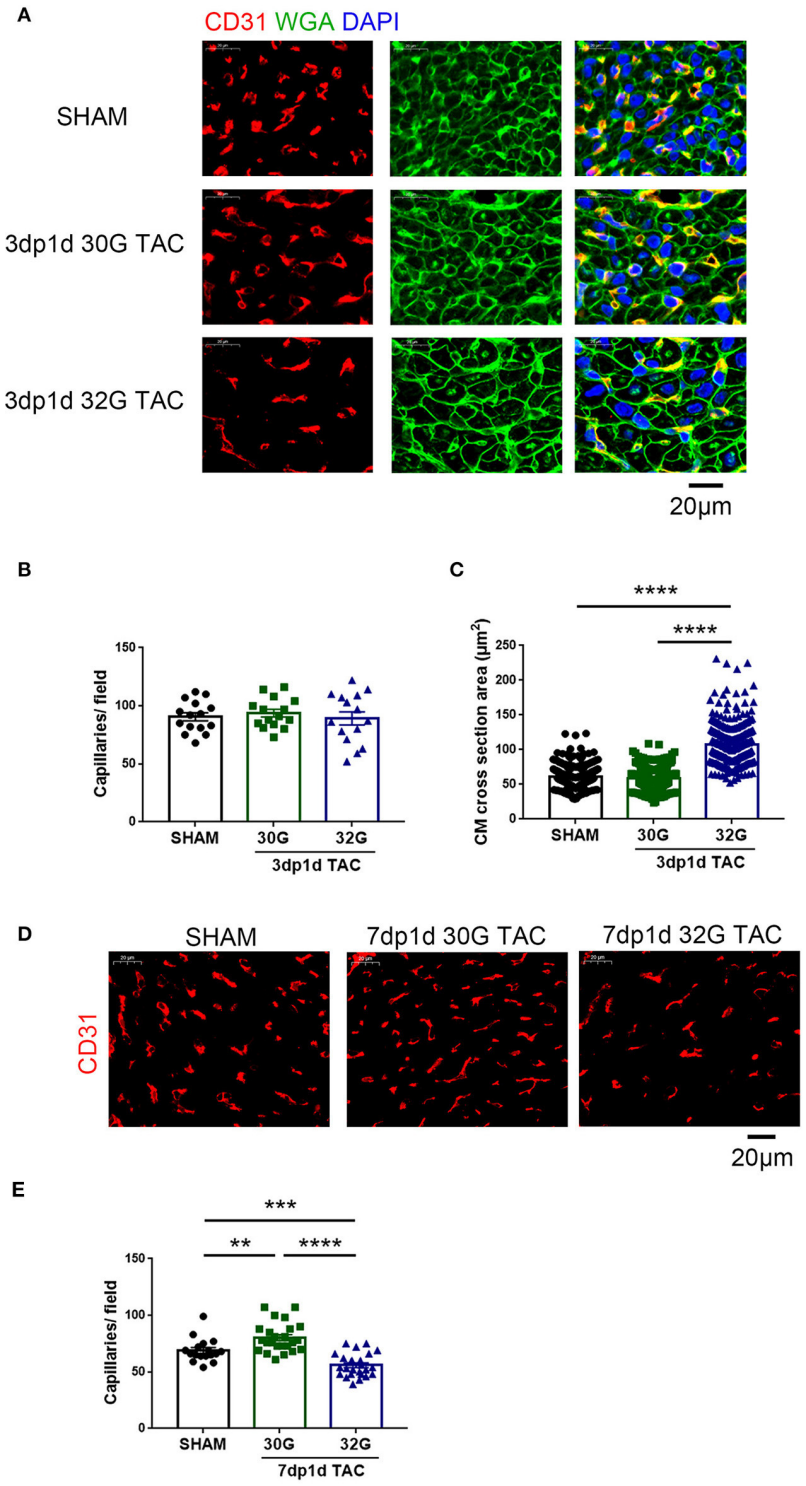
The effect of different constrictions on myocardial hypertrophy and angiogenesis. **(A)** Immunostaining for CD31 (red), wheat germ agglutinin (WGA, green), and DAPI in heart sections. Scale bar: 20 μm. **(B)** Quantification of CD31^+^ capillaries/field; *n* = 3 hearts per group. Each heart slide was random selected with five high-power fields for statistical analysis. **(C)** Analysis of cardiomyocyte cross-sectional area in the three groups; *n* = 3 hearts per group. A total of 300 cardiomyocytes per group were analyzed. **(D,E)** Immunostaining of heart slides for CD31 and quantification of capillary density. Scale bar: 20 μm; *n* = 3 hearts per group. Each heart slide was random selected with five to eight high-power fields for statistical analysis. Data are presented as mean ± SEM. **P < 0.01, ***P < 0.001, and ****P < 0.0001. Statistical significance was calculated using the unpaired two-tailed *t*-test.

Vascular proliferation is necessary for cardiomyocyte proliferation. The vascular tissue was labeled with CD31 to detect whether angiogenesis was activated. No change in capillary density was observed 3 days after TAC (Figures 4A,B). The capillary density increased in mice in the 30G TAC group but decreased in those in the 32G TAC group 7 days after the surgery (Figures 4D,E).

These data indicated that mild pressure overload stimulated cardiomyocyte proliferation and angiogenesis, while moderate pressure overload hampered the expected growth of cardiomyocytes.

### Both Mild and Moderate Pressure Overload Caused Myocardial Hypertrophy in Neonatal Mice

The aforementioned echocardiography results provided a general understanding of myocardial hypertrophy in mice after TAC *in vivo*. Next, the myocardial hypertrophy–related indicators were detected in the isolated hearts.

**Figure 5:**
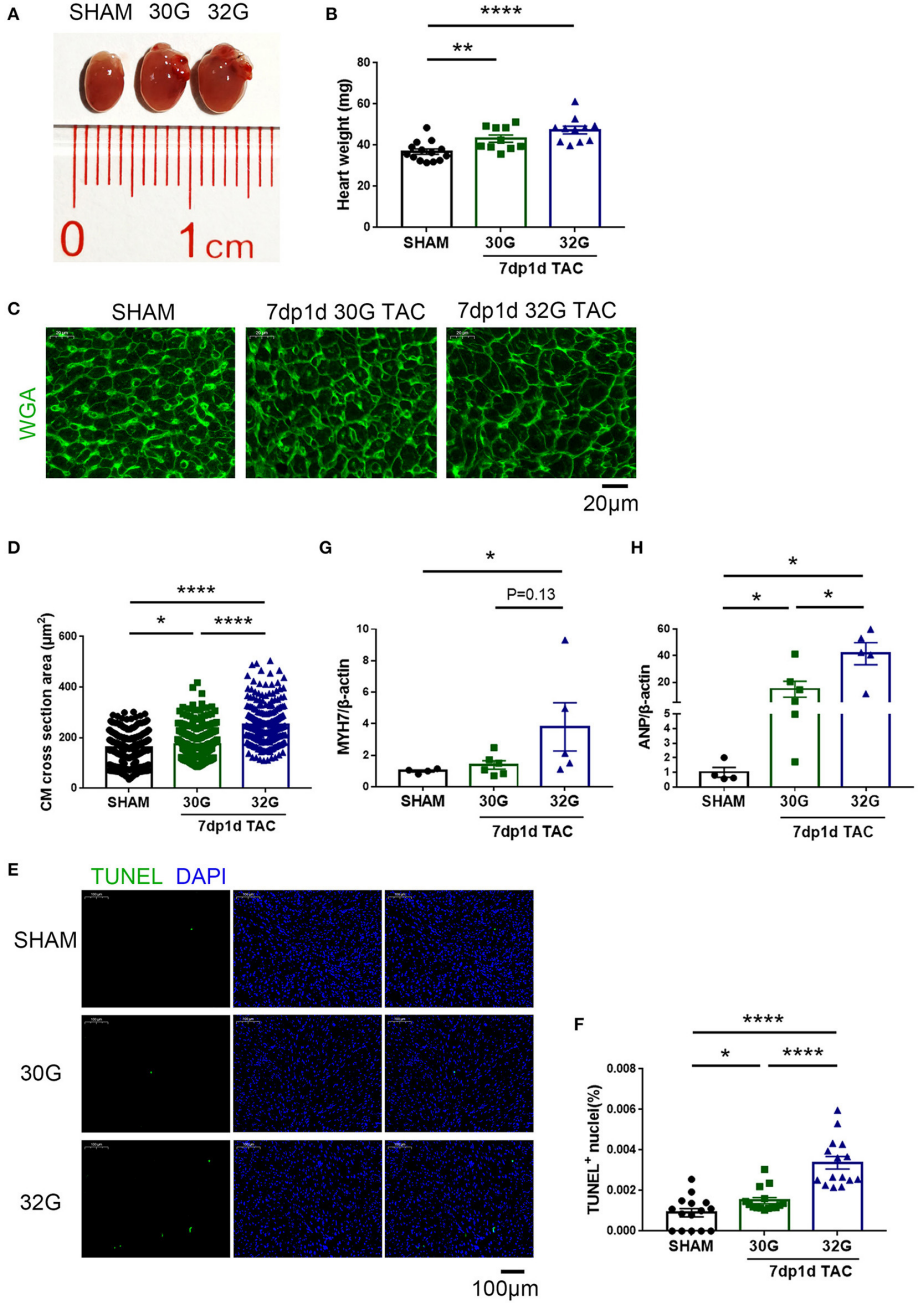
Apoptosis and hypertrophy increased in mice 7 days after the 30G and 32G TAC surgery. **(A)** The hearts of mice in the sham (left), 30G TAC (middle), and 32G TAC (right) groups. **(B)** The heart weight analysis in the three groups: *n* = 14, sham; *n* = 10, 30G TAC; *n* = 11, 32G TAC. **(C)** Representative heart sections stained with WGA (green) in the three groups. Scale bar: 20 μm. **(D)** Analysis of cardiomyocyte cross-sectional area in the three groups; *n* = 3 hearts per group. A total of 250-300 cardiomyocytes per group were analyzed. **(E)** Representative images of terminal deoxynucleotidyl transferase dUTP nick end labeling (TUNEL, green)-positive cells from the heart sections in the three groups. Scale bar: 100 μm. **(F)** Statistical chart of TUNEL-positive cells in the three groups; *n* = 3 hearts per group. Each heart slide was random selected with five high-power fields for statistical analysis. **(G,H)** Quantitative real-time polymerase chain reaction (qRT-PCR) for the expression of myosin heavy chain-β (MYH7) and atrial natriuretic peptide (ANP), respectively, in the ventricular tissue; *n* = 4, sham; *n* = 6, 30G TAC; *n* = 5, 32G TAC. Data are presented as mean ± SEM. *P < 0.05, **P < 0.01, and ****P < 0.0001. Statistical significance was calculated using the unpaired two-tailed *t*-test or Mann–Whitney test.

No evident hypertrophy was found in the 30G TAC group, but prominent hypertrophy was observed in the 32G TAC group 3 days after the surgery (Figures 4A,C). The harvested hearts 7 days after TAC supported the aforementioned echocardiography results. The global heart size and weight increased in the TAC groups compared with that in sham group (Figures 5A,B). The increased cross-sectional area (CSA) of individual cardiomyocytes was associated with increased cardiac tissue weight (Figures 5C,D). The expression of hypertrophic markers (22), myosin heavy chain-β (MYH7), and atrial natriuretic peptide (ANP) was detected by quantitative real-time polymerase chain reaction (qRT-PCR) analysis. The results showed that 32G TAC significantly increased the expression of MYH7 and ANP (Figures 5G,H) in mouse hearts. The expression of MYH7 (*P* = 0.48) and ANP (*P* = 0.02) mildly elevated in the 30G TAC group compared with that in the sham group. Cardiomyocyte apoptosis was determined by TdT-mediated dUTP Nick-End Labeling (TUNEL) staining of the heart sections 7 days after TAC. The number of TUNEL-positive cells was more in the 32G TAC group than in the sham and 30G TAC groups (Figures 5E,F).

**Figure 6:**
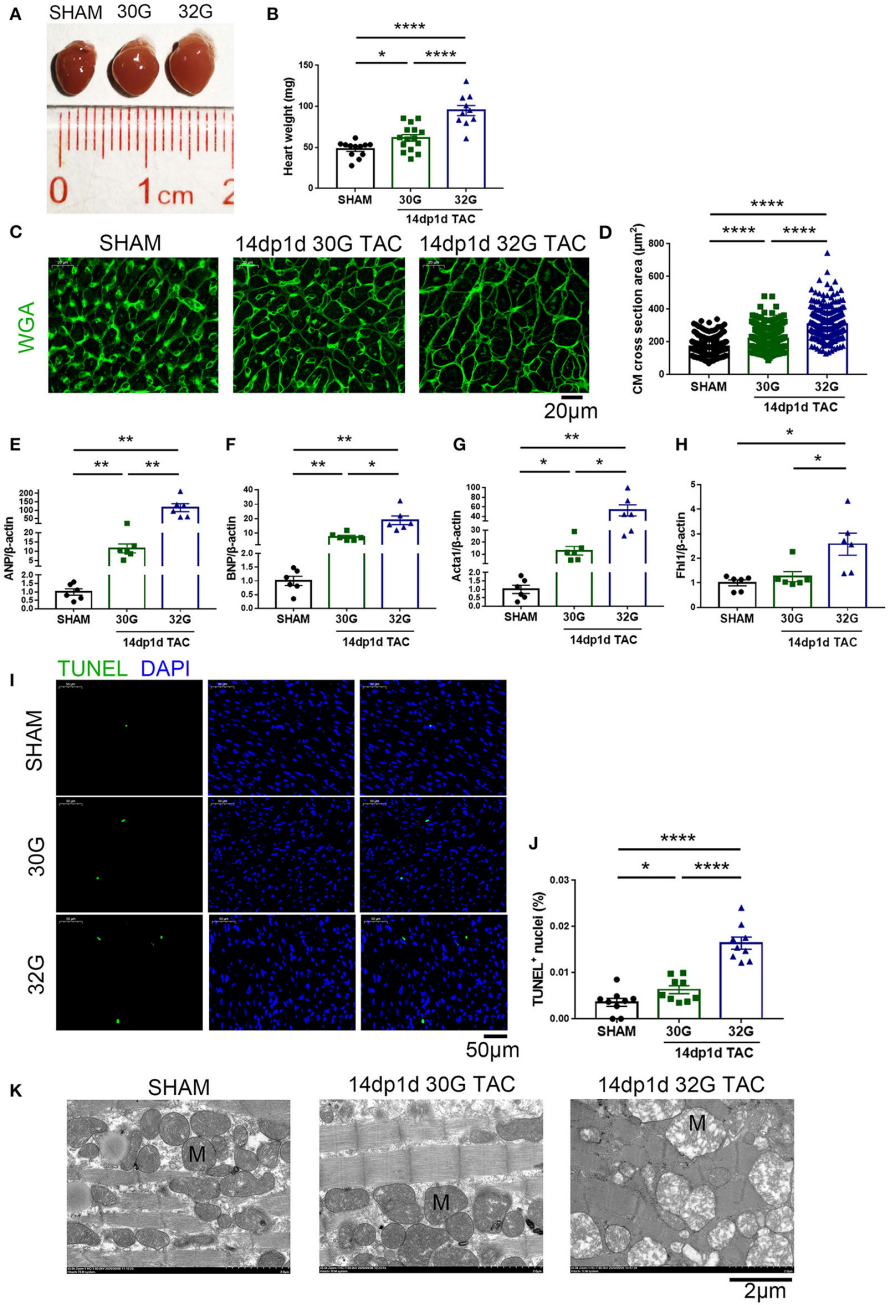
Apoptosis and hypertrophy increased in mice 14 days after the 30G and 32G TAC. **(A)** Hearts of mice in the sham (left), 30G TAC (middle), and 32G TAC (right) groups. **(B)** The heart weight analysis in the three groups: *n* = 12, sham; *n* = 15, 30G TAC; *n* = 10, 32G TAC. **(C)** Representative heart sections stained with WGA (green) in the three groups. Scale bar: 20 μm. **(D)** Analysis of cardiomyocyte cross-sectional area in the three groups; *n* = 3 hearts per group. A total of 300 cardiomyocytes per group were analyzed. **(E–H)** Expression of ANP, brain natriuretic peptide (BNP), Acta1 and four and a half LIM domains 1 (Fhl1) in the ventricular tissue; *n* = 6, sham; *n* = 6, 30G TAC; *n* = 6, 32G TAC. **(I)** Representative images of TUNEL-positive cells from heart sections in the three groups. Scale bar: 50 μm. **(J)** Statistical chart of TUNEL-positive cells in the three groups; *n* = 3 hearts per group. Each heart slide was random selected with three high-power fields for statistical analysis. **(K)** Representative transmission electron microscopy (×5,000) images revealed normal sham heart (left), hypertrophic 30G TAC heart (middle), and 32G TAC heart (right) with hypotrophy and mitochondrial swelling. M, Mitochondria. Scale bar: 2 μm. Data are presented as mean ± SEM. *P < 0.05, **P < 0.01, and ****P < 0.0001. Statistical significance was calculated using the unpaired two-tailed *t*-test.

The heart size and weight of mice increased 14 days after the TAC surgery (Figures 6A,B). The CSA of individual cardiomyocytes increased more in the 32G TAC group than in the 30G TAC group (Figures 6C,D). Furthermore, the expression of hypertrophy biomarkers was higher in the 32G TAC group than in the 30G TAC group. qRT-PCR results showed that the mRNA expression of ANP, brain natriuretic peptide and Acta1 significantly increased in the 30G TAC and 32G TAC groups, and the expression of Fhl1 elevated only in the 32G TAC group (Figures 6E–H). The number of TUNEL-positive cells was higher in the 32G TAC group than in the sham and 30G TAC groups (Figures 6I,J).

### Ultrastructural Alterations in the Hearts of Mice After TAC

The LV structure was analyzed by transmission electron microscopy (TEM, Hitachi, HT7800, Japan) 14 days after TAC to understand further the ultrastructural alterations resulting from mild and moderate pressure overload. Significant sarcomere disarray was displayed in the hearts of mice after 32G TAC (Figure 6K). Crowded mitochondria became irregularly shaped due to mutual extrusion, and hypertrophied mitochondria were observed in the hyperplastic mitochondrial population. The mitochondria became enlarged, the cristae became shorter or disappeared, the matrix density decreased, and the mitochondrial vacuoles were denatured in the 32G TAC group compared with those in the sham group. On the contrary, mice in the 30G TAC group were more likely to have myocardial compensatory manifestations such as cardiomyocyte hypertrophy and mitochondrial hyperplasia (Figure 6K).

### Excessive Pressure Overload Led to Significant Myocardial Fibrosis

**Figure 7:**
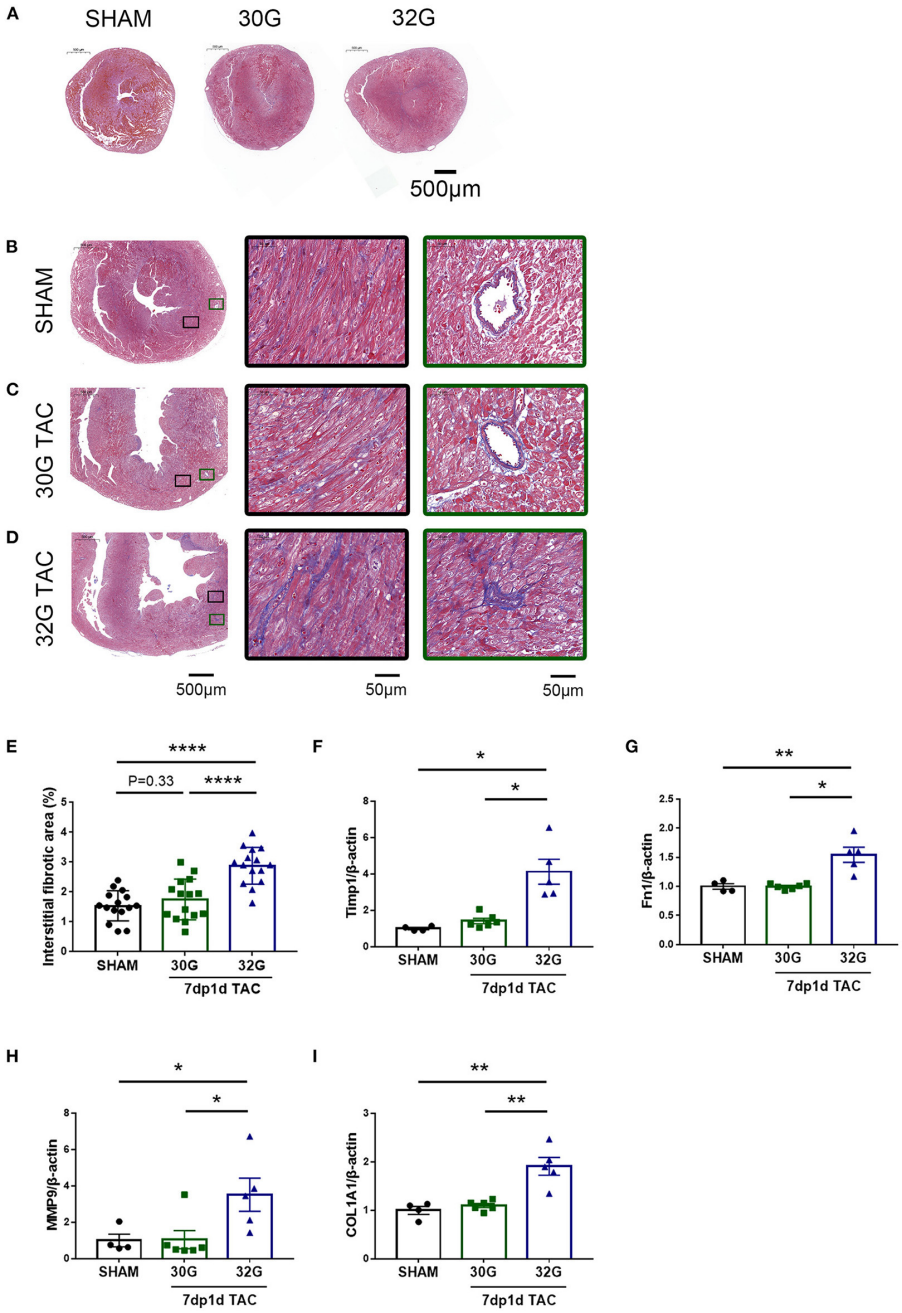
Significantly increased fibrosis in the hearts of mice in the 32G TAC group 7 days after the surgery. **(A)** Masson staining of sham, 30G TAC, and 32G TAC mouse hearts. Scale bar: 500 μm. **(B–D)** Representative Masson trichrome staining in the hearts of mice in the sham **(B)**, 30G TAC **(C)**, and 32G TAC **(D)** groups. The middle images are high-power fields of the corresponding left images (black square frame), showing interstitial fibrosis. The right images are high-power fields of the corresponding left images (green square frame) showing perivascular fibrosis. Scale bar: 500 μm (left images); scale bar: 50 μm (middle and right images). **(E)** Interstitial fibrosis in the three groups; *n* = 3 hearts per group. Each heart slide was random selected with five high-power fields for statistical analysis. **(F–I)** qRT-PCR for the expression of matrix metallopeptidase 9 (MMP9), tissue inhibitor of metalloproteinases 1(Timp1), fibronectin 1(Fn1), and collagen type I alpha 1 chain (COL1A1) in the ventricular tissue; *n* = 4, sham; *n* = 6, 30G TAC; *n* = 5, 32G TAC. Data are presented as mean ± SEM. *P < 0.05, **P < 0.01, and ****P < 0.0001. Statistical significance was calculated using unpaired two-tailed *t*-test or Mann–Whitney test.

Masson staining was performed, and the expression of fibrosis markers was detected to investigate the effect of pressure overload on myocardial fibrosis. No prominent fibrosis was observed 3 days after the surgery (Figure 7A). Noticeable interstitial and perivascular myocardial fibrosis were observed in the 32G TAC group compared with the 30G TAC and sham groups 7 days after the surgery (Figures 7B–E). This finding was confirmed by the qRT-PCR of the cardiac fibrosis–related genes, including matrix metallopeptidase 9 (MMP9), collagen type I alpha 1 chain (COL1A1), fibronectin 1 (Fn1), and tissue inhibitor of metalloproteinases 1 (Timp1). Mice in the 32G TAC group had a higher expression of these genes compared with those in the sham group, while those in the 30G TAC group had no obvious elevation, according to Masson staining (Figures 7F–I).

**Figure 8:**
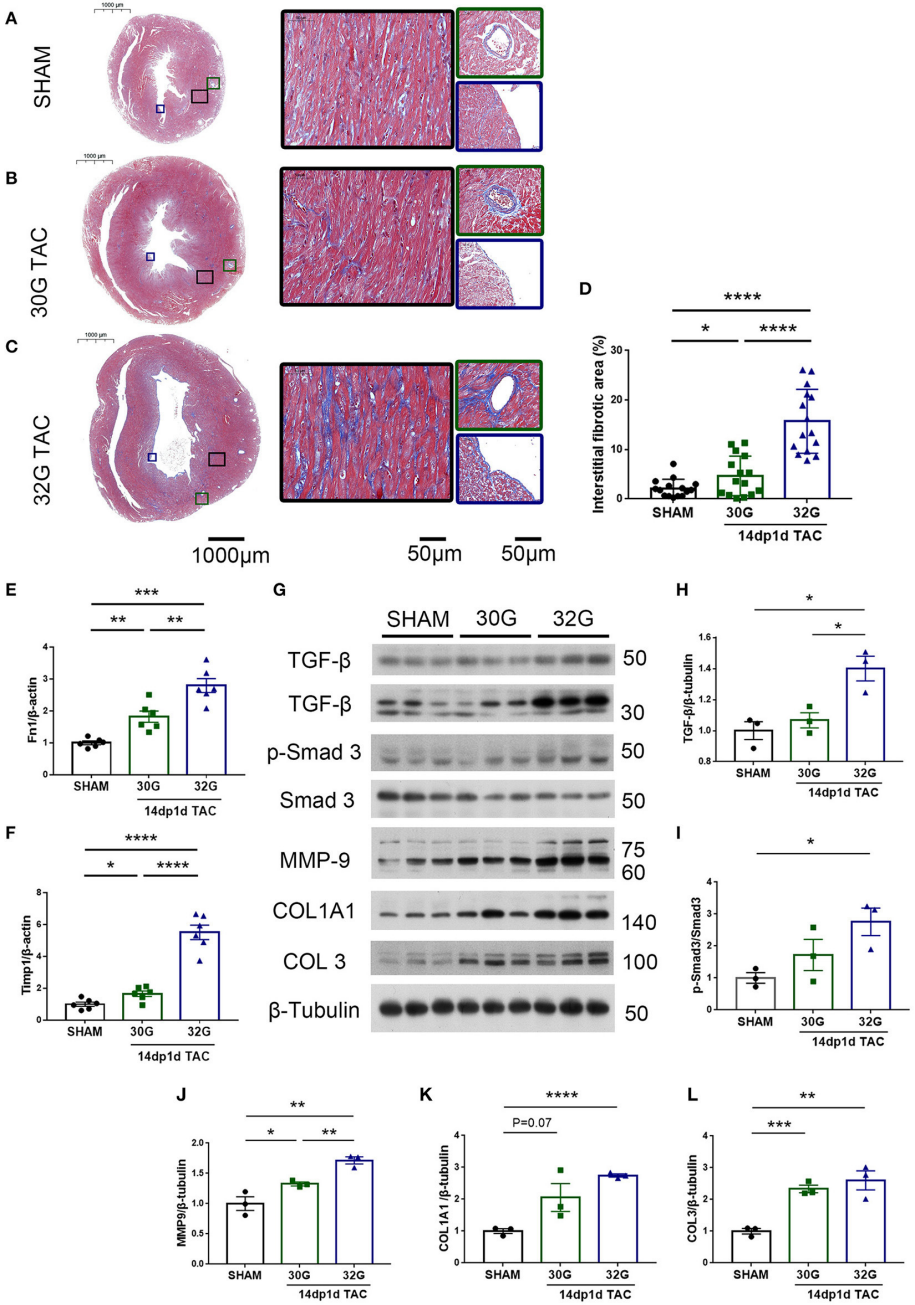
Hearts of mice in the 32G TAC group had more fibrosis markers compared with those in the sham and 30G TAC groups. **(A–C)** Representative Masson staining of hearts of mice in the sham **(A)**, 30G TAC **(B)**, and 32G TAC **(C)** groups. The middle images are high-power fields of the corresponding left images (black square frame), showing interstitial fibrosis. The right images are high-power fields of the corresponding left images, showing perivascular fibrosis (green square frame, up) and endomyocardial fibrosis (blue square frame, down). Scale bar: 100 μm (left images); scale bar: 50 μm (middle and right images). **(D)** Interstitial fibrosis in the three groups; *n* = 3 hearts per group. Each heart slide was random selected with five high-power fields for statistical analysis. **(E,F)** qRT-PCR for the expression of Fn1 and Timp1 in the ventricular tissue; *n* = 6, sham; *n* = 6, 30G TAC; *n* = 6, 32G TAC. **(G)** Western blot stripes from three independent hearts in the three groups are shown. **(H–L)** Quantification of protein expression of TGF-β/β-tubulin **(H)**, p-Smad3/Smad3 **(I)**, MMP-9/β-tubulin **(J)**, COL1A1/β-tubulin **(K)**, and COL3/β-tubulin **(L)** in the hearts of mice in the sham, 30G TAC, and 32G TAC groups 14 days after the surgery. Data are presented as mean ± SEM. *P < 0.05, **P < 0.01, ***P < 0.001, and ****P < 0.0001. Statistical significance was calculated using the unpaired two-tailed *t*-test.

Myocardial fibrosis further increased 14 days after TAC. The fibrotic area increased by 1.2 times in the 30G TAC group and 6.2 times in the 32G TAC group compared with the sham group (Figures 8A–D). The gene expression of Fn1 and Timp1 in the hearts of mice in the 30G TAC and 32G TAC groups further increased (Figures 8E,F). The signal transduction in the myocardial fibrosis–promoting pathway was detected by Western blot analysis (Figure 8G). Excessive pressure overload increased the expression of transforming growth factor-β (TGF-β) and pSmad3/Smad3 in the 32G TAC group (Figures 8H,I). Besides, the expression of extracellular matrix components MMP-9, COL1A1, and COL3 increased significantly (Figures 8J–L). The expression of extracellular matrix proteins increased insignificantly in the 30G TAC group compared with the sham group (Figures 8G–L). Only the expression of MMP-9 and COL3 mildly increased. These results suggested that excessive pressure overload activated TGF-β/Smad3 signaling pathways and led to myocardial fibrosis.

## Discussion

**Figure 9:**
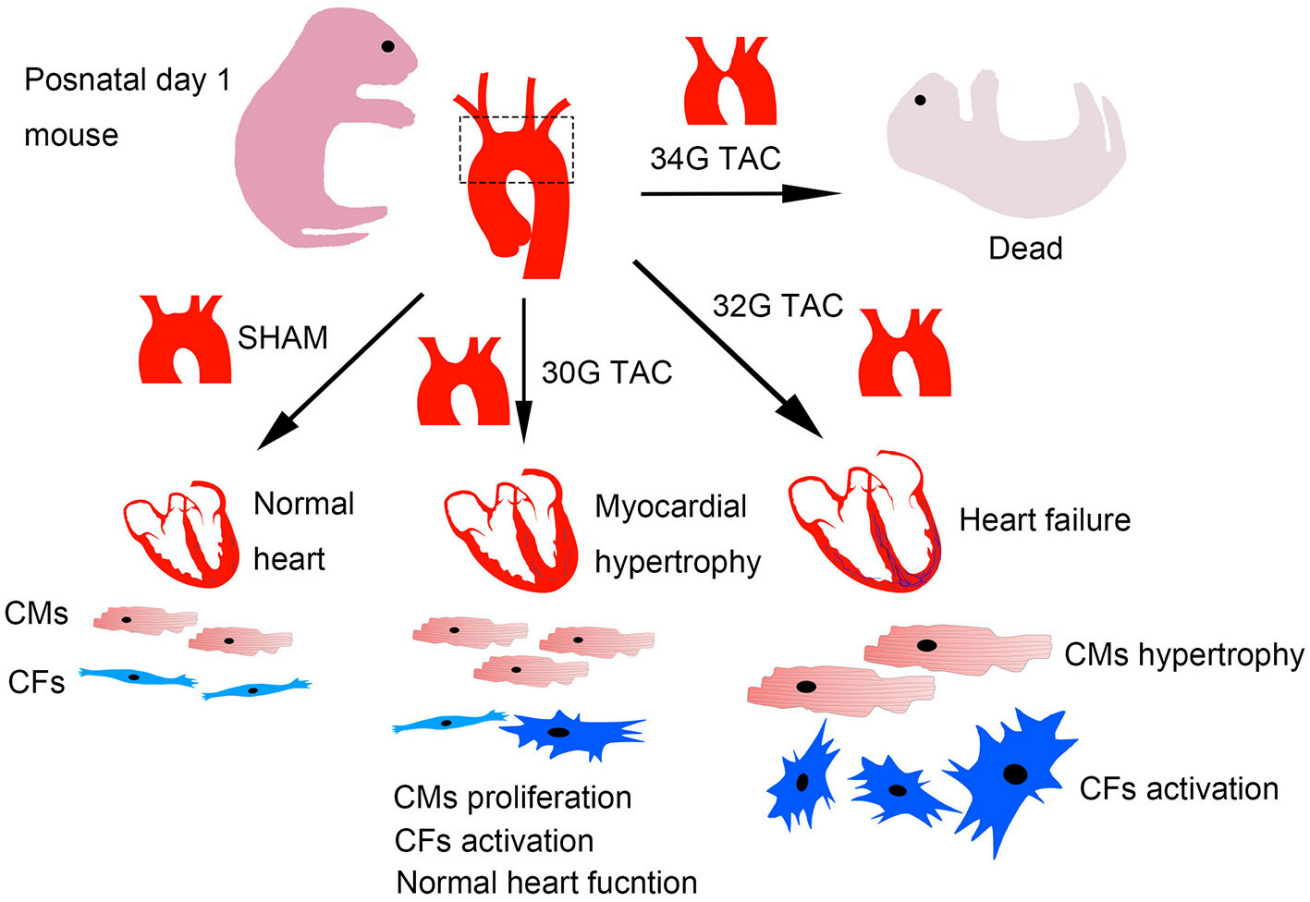
Graphical diagram of the consequences of different pressure overloads in neonatal mice. Cardiomyocytes were effectively stimulated to proliferate under mild pressure overload with a reserved heart function. Moderate heart pressure overload caused cardiomyocyte hypertrophy and fibrosis, further influencing cardiomyocyte proliferation and heart function. The mice barely survived after severe pressure overload.

This study provided data characterizing a mouse model of TAC to better understand the structural and functional changes under pressure overload in neonatal mice. The differences in neonates were analyzed under three pressure overload levels, including heart structure and function, myocardial hypertrophy, and fibrosis. A widely distinct response was detected. The results showed that mild pressure overload effectively stimulated cardiomyocyte proliferation, but moderate pressure overload caused severe cardiac pathological hypertrophy, fibrosis, and heart dysfunction. Further, severe pressure overload led to high mortality in mice after the surgery (Figure 9).

In 2017, Wang et al. (18) found that the progress of right ventricular remodeling was much faster in neonates with pulmonary artery constriction than in adults. Yet, they did not explore whether the regeneration of cardiomyocytes existed. Their findings were similar to the results in the present study on 32G TAC surgery, despite using different strains of animals and surgical methods. The proliferated cardiomyocytes were seldom detected and the heart function was severely affected 7 days after the surgery. The increased myocardial fibrosis and hypertrophy indicated that the hearts of 32G TAC mice developed pathological myocardial remodeling following cardiac decompensation (23). These results indicated that excessive pressure overload could significantly affect the heart structure and function, further threatening the survival of the pups.

The promotion of cardiomyocyte proliferation with ascending aortic constriction in neonatal rats was first reported by Wang et al. (19). Their findings were similar to the results of the present study on 30G TAC surgery in mice. The increased cardiomyocyte proliferation was found at 3 and 7 day after surgery and the heart function was not affected. The results indicated that the heart with proliferated cardiomyocytes could fight against mild pressure overload effectively. The relative constriction degree increased with growth in mice. The corrected LV mass showed a significant increase 14 days after the surgery, which might be caused by the proliferation and adaptive hypertrophy of cardiomyocytes.

The TAC model of neonatal mice has been studied in 2019 (20). The researchers bound the transverse aorta with a 33G needle, which further promoted cardiomyocyte proliferation and angiogenesis without myocardial fibrosis and hypertrophy 14 days after the surgery. These results were different from those of the present study. In the present study, 32G (0.23 mm) TAC, which had a lower constriction degree than 33G (0.21 mm) TAC, caused severe heart failure in the 7dp1d TAC and 14dp1d TAC subgroups. Their findings more like the positive changes in 14dp1d 30G TAC in the present study. The following reasons might explain the discrepancy. First, different strains of mice were used. The ICR-CD1 and C57BL/6J mice may have a different diameter of the aortic arch, implying no comparability in terms of the degree of constriction even if the same-gauge needle was used. Second, the growth rate of the two strains might be different. A faster weight gain might result in worse heart structure and function after the surgery. Finally, the tightness of knots during the surgery was vital. Only tight knots between the aortic arch and the needle could ensure the study's consistency and repeatability. They also found that TAC on postnatal day 7 caused heart dysfunction, myocardial hypertrophy, and fibrosis without cardiomyocyte proliferation. Consistent with these results, the present study found severe heart failure in mice in the 7dp8d 30G TAC subgroup, but the change in myocardial hypertrophy and fibrosis was similar to that in the 14dp1d 30G TAC subgroup (data not shown).

Many factors take part in heart regeneration. The stiffness of the microenvironment is a new mechanism (24). Mario and his team found no heart regeneration due to the changes in the structure of the extracellular matrix and cytoskeleton on postnatal days 1 and 2. In the present study, the excessive pressure overload induced by 32G TAC caused changes in extracellular matrix components, detrimental to cardiomyocyte proliferation and compensation. However, an appropriate elevated pressure could stimulate cardiomyocyte proliferation without significant changes in the extracellular matrix. The inactive of cardiomyocytes proliferation partly result from the stiffness of the microenvironment.

Gradually, pressure overload on neonates has gained the attention of researchers. Ye et al. (25) performed an RNAseq analysis on cardiomyocytes from the right ventricle of neonatal mice with pulmonary artery banding and reported differential expression of genes that mainly mediated mitosis and cell division. Consistent with their point of view, the present study also considered that pressure overload in neonates was better than apex resection and MI models to study cardiomyocyte proliferation. Heart regeneration in the apex resection model was questioned by Andersen et al. (26). Their results showed fibrotic scar and collagen disposition at the resection border instead of proliferated cardiomyocytes. Li et al. (27) revealed that the cutting length and angle were essential to the stability of regenerative capacity, which needed much practice. On the contrary, TAC had better repeatability and clinical value compared with apical resection in neonates.

Mice with different pressure overload can serve as models for different severity and clinical phenotypes of congenital aortic coarctation from a translational perspective. They can be used to study the pathological changes in patients with CoA and provide adequate medical treatment besides surgery. Meanwhile, 7dp1dTAC with 32G constriction and 7dp8dTAC with 30G constriction are fantastic ways to study heart failure due to the shortened experimental period.

The level of pressure overload determines heart regeneration or failure. It is essential to choose an appropriate constriction degree for the study according to the mouse strains; otherwise, the desired results may not be obtained. In conclusion, this study showed that different levels of pressure overload resulted in radically different consequences in neonatal mice using a standard procedure of TAC. The proliferation of cardiomyocytes was promoted under pressure overload were confirmed, but some issues remained. Patients with CoA usually have extensive lateral vessel formation (28). Whether the development of collateral circulation participated in the normal heart function in the 30G TAC group was unknown. Whether the proliferating cells were derived from cardiac stem cells or other cells with differentiation potential was also not confirmed. The similarities and differences of factors that promote cardiomyocyte proliferation between pressure overload and MI surgery remain to be studied. The findings of this study might have important implications for the exploration of myocardial regeneration. Further research on the TAC model will provide valuable help to myocardial hypertrophy in adults.

## Methods

### Mice

The C57BL/6J mice were acquired from Guangdong Medical Laboratory Animal Center (Guangzhou, China) and housed in a temperature-controlled environment with 12-h light/dark cycles and food and water available ad libitum. Postnatal day 1 (P1) mice of both sexes were used in this study.

After 3 and 7 days of surgery, the mice were sacrificed by putting them on an ice water bath for 5–10 min until movement stopped and respiration ceased. The mice were euthanized by cervical dislocation after anesthetized with 2.0% isoflurane inhalation 14 days after surgery.

All animal protocols followed the Guidelines for the Care and Use of Laboratory Animals published by the US National Institutes of Health (8th edition, 2011). All animal procedures were performed under the guidelines of Guangzhou Medical University Animal Research Committee (Guangzhou, China; permit number: A2020-008).

### TAC in Neonatal Mice

TAC surgery was performed on P1 mice (Supplementary Materials). The neonates were anesthetized by hypothermia in an ice water bath for about 4 min when the skin turned white and the movement stopped. Hypothermia was accompanied by apnea and cardiac arrest, preventing excessive blood loss during the surgery. The neonates were transferred to the ice bed to remain anesthetized in the supine position and immobilized by taping the arms, legs, and head.

The following operation was performed under a microscope (RWD, 77001S, China). The skin was cut longitudinally from the neck, and a midsternal incision across the neck to the second rib was made with fine surgical scissors. Next, the submandibular gland and sternothyroid muscle were separated by blunt dissection to visualize the sternum stem and cut the sternum longitudinally from the neck down the median line to the second rib. The left and right lobes of the thymus were separated with a self-made pull hook to expose the aortic arch, and a 10–0 suture was inserted under the transverse aorta with a microneedle holder between the brachiocephalic trunk and the left common carotid artery.

Separately, a 30G, 32G, or 34G needle was placed in parallel to the transverse aorta, three knots was tied around the transverse aorta and the needle tightly, and then the needle was removed gently. The sternum and skin were orderly closed with an 8–0 nonabsorbable prolene suture. The pups were warmed up by putting them on a 37°C heating pad. Their bodies turned red, and they recovered breathing and normal motion within 15 min. The mice returned to their foster mothers and mixed with their littermates as soon as possible, which enhanced mouse survival. The same procedures were performed without aorta constriction in littermates serving as the sham-operated group.

The TAC performed on P1 was defined as 1d TAC and subsequently observed 3, 7, 14, and 21 days after TAC (3dp1d TAC, 7dp1d TAC, 14dp1d TAC, and 21dp1d TAC, respectively).

### Echocardiography

The LV systolic function was measured 7, 14, and 21 days after TAC with echocardiography using an M-mode echocardiogram Vevo 2100 (VisualSonics, Toronto, Canada). Before the echo procedure, the mice were anesthetized with 2.0% isoflurane to be immobilized. Due to a severe heart rate decrease after inhalation of isoflurane, the mice were not anesthetized during echocardiography. The parasternal short-axis view was used, and the left ventricle recordings were obtained at the papillary muscle level. The measurements of the left ventricle included LV end-diastolic dimension, LV end-systolic dimension, LV posterior wall thickness, and LV anterior wall thickness. In the images we recorded, four boundary lines were drawn along the endocardium and epicardial of the anterior and posterior wall to display the motion trajectory. The EF, FS, corrected LV mass (Corr. LV mass), and other function parameters were calculated using the LV Trace measurement module of Vevo LAB (version 3.1.1) offline analysis software.

To observe the aortic arch, we placed the probe next to the sternum's right side. The probe angle was adjusted to display the long axis view of the aortic arch and the maximum inner diameter of the arch top. The images were collected under M-Mode. Other Artery measurement module in Vascular Package was used to measure the systolic and diastolic vessel inner diameters.

### 5-Bromo-2-Deoxyuridine Pulse-Chase Labeling Experiments

For BrdU labeling experiments, 100 μg/g BrdU (Sigma–Aldrich, B5002, USA) in sterile 0.9% saline was administered subcutaneously to the neonates right after the surgery. The hearts were collected and sectioned 3 days after TAC. For BrdU staining, the heart sections were incubated with anti-BrdU mAb (Servicebio, GB12051, China) to reveal BrdU incorporation. The detailed steps refer to the following immunostaining analysis.

### Histological Analysis

The histology was performed as described previously (29). The hearts were washed with phosphate-buffered saline (PBS), fixed with 4% (*w*/*v*) paraformaldehyde for more than 24 h in room temperature and put in dehydration boxes. The dehydration box was dehydrated with gradient alcohol, got wax leaching, and embedded in wax block. The modified tissue chip wax block was sliced on paraffin slicer (Leica Instrument Company, RM2016, Shanghai), the slice thickness was 2μm. The slice was flattened when floated on the 40°C warm water of the tissue spreader (Kehua Instrument Company, KD-P, Zhejiang), and the tissue was picked up by the glass slides and baked in the oven at 60°C. After the water-baked dried wax was melted, it was taken out and stored at room temperature.

After paraffin removal, 2 μm slide was stained with the Masson trichrome staining method using standard procedures. The slides were visualized using a Pannoramic MIDI Scanner (3DHISTECH Ltd., Budapest, Hungary). Five images were taken from each slide and analyzed using the ImageJ software (National Institutes of Health, MD, USA).

### Immunostaining Analysis

Paraffin-embedded sections were sequentially deparaffinized, rehydrated, antigen retrieved, blocked, and stained first with primary antibodies against phospho-histone 3 (Ser10) (Cell Signaling Technology, 3377, USA) and Ki67 (Abcam, ab16667, USA) at 4°C overnight. The next day, the slides were washed with PBS and incubated with secondary antibody for 50 min at room temperature. The slides were washed with PBS, incubated with diluted tyramide signal amplification—fluorescein isothiocyanate (TSA-FITC) solution (Servicebio, G1222, China) for 10 min in the dark, and then washed with tris buffered saline tween (TBST). The slides were immersed in ethylenediaminetetraacetic acid (EDTA) antigen retrieval buffer and treated in a microwave. Next, they were incubated with the second primary antibody against cardiac troponin I (Proteintech, 21652-1-AP, China) or α-actinin (Servicebio, GB111230, China) overnight at 4°C. On the second day, the slides were washed with PBS and incubated with the secondary antibody for 50 min at room temperature. The slides were stained with 4′,6-diamidino-2-phenylindole (DAPI, Sigma) for 10 min. After removing the liquid, the slides were incubated with a spontaneous fluorescence quenching reagent for 5 min and washed under flowing water for 10 min. Finally, the fluid was removed, and the slides were mounted with an anti-fade mounting medium (Servicebio, G1401).

For wheat germ agglutinin (WGA) staining, the slides were deparaffinized, rehydrated, antigen retrieved, and then incubated for 30 min at 30°C with a primary antibody against WGA conjugated to FITC (Sigma). The slides were stained with DAPI for 10 min and incubated with a spontaneous fluorescence quenching reagent for 5 min. Finally, the liquid was removed, and the slides were mounted with an anti-fade mounting medium. CD31 (Servicebio, GB11063, China) staining went through similar method except DAPI staining.

All slides were visualized using a Pannoramic MIDI Scanner. Images were taken from each slide and analyzed using the Image-Pro Plus 6.0 software (Media Cybernetics, MD, USA).

### Terminal Deoxynucleotidyl Transferase dUTP Nick End Labeling Assay

The heart was harvested at 7 and 14 days after TAC. Apoptotic cardiomyocytes were detected by a TUNEL Assay Kit (Roche) according to the manufacturer's instructions as previous described (30).

### Western Blot Analysis

Western blot analysis was performed as described in a previous study (31). Briefly, the ventricular heart tissue was collected and dissected 7 and 14 days after TAC. The protein was extracted using radio immunoprecipitation assay (RIPA) reagent containing phenylmethanesulfonyl fluoride (PMSF) and phosphatase inhibitors. The protein was separated using 8–10% sodium dodecyl sulfate–polyacrylamide gel electrophoresis and subsequently transferred onto polyvinylidene difluoride membranes (Millipore, IPVH00010, Ireland). After 1 h of blocking with nonfat milk (Bio-Rad, 1706404, USA), the membranes were incubated with primary antibodies at 4°C overnight. The next day, the membranes were washed with TBST and incubated with corresponding horseradish peroxidase (HRP)-conjugated secondary antibodies for 1 h at room temperature. Ultimately, these strips were washed three times with TBST and visualized using an enhanced chemiluminescence kit (Santa Cruz Biotechnology Inc., USA). The antibodies used for Western blot analysis in the study were as follows: anti-Col1A1 (1:250, 91144S, Cell Signaling Technology), anti-TGF-β (1:1,000, 3711S, Cell Signaling Technology), anti-Smad3 (1:1,000, Cell Signaling Technology, 9523S), anti-phospho-Smad3 (1:1,000, 9520S, Cell Signaling Technology), anti-collagen III (1:1,000, Affinity, AF0136, China), anti-MMP9 (1:1,000, Servicebio, GB12132-1, China), anti-β-tubulin (1:5,000, Fdbio Science, FD0064, China), anti-GAPDH (1:5,000, Bioworld, A0063, China), anti-rabbit immunoglobulin (IgG; 1:5,000, Cell Signaling Technology, 7074S), and anti-mouse IgG (1:5,000, Cell Signaling Technology, 7076S).

### Quantitative Real-Time Polymerase Chain Reaction

Ventricular heart tissues were collected and dissected 7 and 14 days after TAC. Total RNA was extracted from tissues using a TRIzol reagent following the manufacturer's protocols (Invitrogen, USA), as described in a previous study (32). For complementary DNA synthesis, 500 ng RNA was reverse transcribed using a PrimeScript RT Reagent Kit (TaKaRa, RR037A, China). qRT-PCR was performed with a TB Green qPCR Mix Kit on an qRT-PCR detection system (Roche, LightCycler 480 II, Switzerland). β-Actin was used as an endogenous control gene to standardize gene expression by the ΔΔCt method. All the primer sequences for qPCR are listed in Supplementary Table 4.

### Transmission Electron Microscopy Imaging

TEM imaging was performed as previously described (33). In brief, the heart was harvested and cut into 1 mm^3^ in the TEM fixative (Servicebio, G1102, China). Next, the tissue block was sequentially fixated, dehydrated, embedded, polymerized, sectioned and stained. Finally, the cuprum grid was observed under TEM (Hitachi, HT7800, Japan) and took images.

### Statistical Analysis

All data were presented as mean value ± standard error of the mean (SEM). Statistical significance was calculated using the unpaired two-tailed *t*-test with SPSS 26.0 statistical software (IBM Corporation, NY, USA) if the data from two groups passed the Levene's test for the equality of variances. Otherwise, the two groups were compared using the unpaired two-tailed *t-*test with Welch's correction or the Mann–Whitney test. A *P* < 0.05 indicated a statistically significant difference (^*^P < 0.05, ^**^P < 0.01, ^***^P < 0.001, and ^****^P < 0.0001).

## Data Availability Statement

The original contributions presented in the study are included in the article/Supplementary Material, further inquiries can be directed to the corresponding author/s.

## Ethics Statement

The animal study was reviewed and approved by Guangzhou Medical University Animal Research Committee.

## Author Contributions

NL and SLiu designed the experiments. JG, XC, YJ, ML, QX, XL, ZL, and SLin performed the experiments. JG, NL, and SLiu wrote the manuscript. All authors read and approved the final manuscript.

## Conflict of Interest

The authors declare that the research was conducted in the absence of any commercial or financial relationships that could be construed as a potential conflict of interest.
