# Supplementary material for: A Neonatal Mouse Model for Pressure Overload: Myocardial Response Corresponds to Severity
Source: Front Cardiovasc Med. 2021 May 21;8:660246. doi: 10.3389/fcvm.2021.660246 (PMC8175619; doi:10.3389/fcvm.2021.660246)
Supplement: Supplementary file 1 [file Data_Sheet_1.docx]

Supplementary Material

# Supplementary Tables

**Supplementary Table S1**

Cardiac function in SHAM, 30G TAC, and 32G TAC mice at 7 days after surgery.

| P8 | SHAM | 30G TAC | 32G TAC |
| --- | --- | --- | --- |
| HR, b.p.m. | 480.88±21.37 | 436.86±14.20 | 392.28±12.98*** # |
| Corr.LV mass, mg | 15.50±0.87 | 19.56±1.49* | 31.52±2.68**** ### |
| EF, % | 83.78±1.89 | 84.11±2.87 | 26.09±4.54**** #### |
| FS, % | 50.27±2.15 | 51.74±3.09 | 11.89±2.24**** #### |
| LVEDD, mm | 1.86±0.06 | 1.76±0.08 | 3.11±0.16**** #### |
| LVESD, mm | 0.93±0.05 | 0.86±0.09 | 2.76±0.0.20**** #### |
| LVPW diastole, mm | 0.51±0.03 | 0.64±0.03* | 0.47±0.04## |
| LVPW systole, mm | 0.84±0.03 | 1.01±0.04** | 0.57±0.05*** #### |
| LVAW diastole, mm | 0.50±0.02 | 0.66±0.04** | 0.49±0.04# |
| LVAW systole, mm | 0.88±0.04 | 0.95±0.04 | 0.58±0.05*** #### |
| LVEDV, μl | 10.69±0.82 | 9.51±1.15 | 39.78±5.07*** ### |
| LVESV, μl | 1.76±0.29 | 1.71±0.46 | 30.86±5.55*** ### |
| SV, μl | 8.94±0.67 | 7.80±0.80 | 8.93±0.91 |
| CO, mL/min | 4.32±0.40 | 3.36±1.00 | 3.54±0.40 |

P8, postnatal day 8; HR, heart rate; LV, left ventricle; EF, ejection fraction; FS, fractional shortening; LVEDD, left ventricular end-diastolic dimension; LVESD, left ventricular end-systolic dimension; LVPW, left ventricular posterior wall thickness; LVAW, left ventricular anterior wall thickness; LVEDV, left ventricular end-diastolic volume; LVESV, left ventricular end-systolic volume; SV, stroke volume; CO, cardiac output; TAC, transverse aortic constriction; n= 7 sham; n= 11 30G TAC; n= 10 32G TAC.

Data are presented as mean ± SEM *P<0.05, **P<0.01, ***P<0.001, ****P<0.0001 vs. SHAM or #P<0.05, ##P<0.01, ###P<0.001, ####P<0.0001 vs. 30G TAC; statistical significance was calculated using unpaired two-tailed t-test.

**Supplementary Table S2**

Cardiac function in SHAM, 30G TAC, and 32G TAC mice at 14 days after surgery.

| P15 | SHAM | 30G TAC | 32G TAC |
| --- | --- | --- | --- |
| HR, b.p.m. | 617.04±39.47 | 542.90±25.82 | 455.59±27.71** # |
| Corr.LV mass, mg | 34.60±2.82 | 55.22±2.20**** | 74.11±7.81** # |
| EF, % | 78.70±3.75 | 76.35±2.77 | 19.49±2.23**** #### |
| FS, % | 49.50±1.80 | 40.98±5.48 | 10.11±0.98**** #### |
| LVEDD, mm | 2.50±0.13 | 2.36±0.09 | 3.64±0.17*** #### |
| LVESD, mm | 1.34±0.13 | 1.33±0.08 | 3.34±0.19**** #### |
| LVPW diastole, mm | 0.64±0.02 | 0.94±0.03**** | 0.76±0.06 # |
| LVPW systole, mm | 1.12±0.05 | 1.34±0.05* | 0.84±0.07** #### |
| LVAW diastole, mm | 0.69±0.03 | 0.98±0.04**** | 0.73±0.07 ## |
| LVAW systole, mm | 1.08±0.04 | 1.43±0.05*** | 0.83±0.07* #### |
| LVEDV, μl | 22.95±2.73 | 19.58±1.71 | 57.38±7.02*** ### |
| LVESV, μl | 5.05±0.1.31 | 4.62±0.65 | 46.87±6.78*** ### |
| SV, μl | 17.91±2.13 | 14.97±1.36 | 10.52±0.0.93* # |
| CO, mL/min | 11.21±1.73 | 8.18±0.91 | 4.87±0.0.58** ## |

P15, postnatal day 15; n= 7 sham; n= 8 30G TAC; n= 8 32G TAC.

Data are presented as mean ± SEM *P<0.05, **P<0.01, ***P<0.001, ****P<0.0001 vs. SHAM or #P<0.05, ##P<0.01, ###P<0.001, ####P<0.0001 vs. 30G TAC; statistical significance was calculated using unpaired two-tailed t-test.

**Supplementary Table S3**

Cardiac function in SHAM and 30G TAC mice at 21 days after surgery.

| P22 | SHAM | 30G TAC |
| --- | --- | --- |
| HR, b.p.m. | 593.09±38.66 | 625.46±37.44 |
| Corr.LV mass, mg | 35.37±5.65 | 56.21±4.83* |
| EF, % | 78.70±3.75 | 76.35±2.77 |
| FS, % | 45.26±2.22 | 48.71±3.86 |
| LVEDD, mm | 2.71±0.10 | 2.92±0.09 |
| LVESD, mm | 1.49±0.11 | 1.50±0.13 |
| LVPW diastole, mm | 0.56±0.06 | 0.76±0.03* |
| LVPW systole, mm | 1.03±0.09 | 1.31±0.09 |
| LVAW diastole, mm | 0.67±0.08 | 0.82±0.05 |
| LVAW systole, mm | 1.05±0.10 | 1.34±0.09* |
| LVEDV, μl | 27.72±2.65 | 32.97±2.55 |
| LVESV, μl | 6.43±0.1.26 | 6.63±1.31 |
| SV, μl | 21.29±1.48 | 26.34±1.94 |
| CO, mL/min | 12.39±0.66 | 16.20±1.02** |

P22, postnatal day 22; n= 7 sham; n= 7 30G;

Data are presented as mean ± s.e.m. *P<0.05 vs. SHAM; statistical significance was calculated using unpaired two-tailed t-test.

**Supplementary Table S4**. Primer sequence for qRT-PCR.

| Gene | Forward sequence | Reverse sequence |
| --- | --- | --- |
| Col1a1 | TCCTGACGCATGGCCAAGAAGACA | TCCGGGCAGAAAGCACAGCACTC |
| Col3a1 | GCACAGCAGTCCACCGTAGA | TCTCCAAATGGGATCTCTGG |
| MMP9 | CAAAGACCTGAAAACCTCCAAC | GACTGCTTCTCTCCCATCATC |
| Timp1 | ATATCCGGTACGCCTACACC | GCCCGTGATGAGAAACTCTT |
| Fn1 | ATGTGGACCCCTCCTGATAGT | GCCCAGTGATTTCAGCAAAGG |
| ANP | GTGTACAGTGCGGTGTCCAA | ACCTCATCTTCTACCGGCATC |
| BNP | ACAAGATAGACCGGATCGGA | AGCCAGGAGGTCTTCCTACA |
| Acta1 | AGGCGGTGCTGTCTCTCTAT | GACATTGTGGGTGACACCAT |
| Fhl1 | ACTGCGTGGATTGCTACAAG | TTTACCAAACCCAGTGATGG |
| Myh7 | ATGTGCCGGACCTTGGAAG | CCTCGGGTTAGCTGAGAGATCA |
| β-actin | CTACCTCATGAAGATCCTGACC | CACAGCTTCTCTTTGATGTCAC |
